# In vitro evaluation of gentamicin activity against Spanish field isolates of *Brachyspira hyodysenteriae*

**DOI:** 10.1186/s40813-022-00291-w

**Published:** 2022-12-01

**Authors:** Clara Vega, Lucía Pérez-Pérez, Héctor Argüello, Manuel Gómez-García, Héctor Puente, Ignacio Fernández-Usón, Pedro Rubio, Ana Carvajal

**Affiliations:** 1 Department of Animal Health, Faculty of Veterinary Medicine, Universidad de León, Campus de Vegazana S/N, 24007 León, Spain; 2 INDEGSAL, Universidad de León, Campus de Vegazana S/N, 24007 León, Spain; 3 Fatro Ibérica S.L., C/ Constitución 1 PB3, Sant Just Desvern, 08960 Barcelona, Spain

**Keywords:** Spirochaetes, Antibiotic, Aminoglycoside, Swine dysentery, Antimicrobial susceptibility testing, Resistance

## Abstract

**Background:**

The treatment of swine dysentery (SD) has become constrained in recent years due to the limited availability of effective drugs combined with a rise in antimicrobial resistance. Gentamicin, an aminoglycoside antibiotic, is authorised for the control of this disease in several European countries but has not been extensively used so far. In this study, the in vitro susceptibility of 56 *Brachyspira hyodysenteriae* field isolates was evaluated against gentamicin using a broth microdilution test. The molecular basis of decreased susceptibility to gentamicin was also investigated by sequencing the 16S rRNA gene and phylogenetic relatedness by multiple-locus variable number tandem-repeat analysis (MLVA).

**Results:**

Most *B. hyodysenteriae* isolates presented low minimum inhibitory concentration (MIC) values to gentamicin, with a mode of 2 µg/mL, a median or MIC_50_ of 4 µg/mL and percentile 90 or MIC_90_ of 16 µg/mL. The distribution of these values over the period studied (2011–2019) did not show a tendency towards the development of resistance to gentamicin. Differences in susceptibility among isolates could be explained by two point-mutations in the 16S rRNA gene, C990T and A1185G, which were only present in isolates with high MICs. These isolates were typed in three different MLVA clusters. Analyses of co-resistance between gentamicin and antimicrobials commonly used for the treatment of SD revealed that resistance to tiamulin and valnemulin was associated with low MICs for gentamicin.

**Conclusions:**

The results provide an accurate characterisation of antimicrobial sensitivity to gentamicin and possible mechanisms of resistance in Spanish *B. hyodysenteriae* isolates. These findings allow us to propose gentamicin as an alternative in the antibiotic management of SD, particularly in outbreaks caused by pleuromutilin resistant isolates.

**Supplementary Information:**

The online version contains supplementary material available at 10.1186/s40813-022-00291-w.

## Background

SD is a common enteric disease among pigs worldwide mainly occurring during the growing and finishing periods, which causes important losses arising from increased mortality, reduced feed conversion and growth and treatment expenses [1]. Although *Brachyspira hyodysenteriae* was the classically recognized aetiological agent of SD, it has been demonstrated that other strongly β-haemolytic spirochaetes such as *Brachyspira hampsonii* and *Brachyspira suanatina* also cause SD [2]. These anaerobic bacterial species colonize the large intestine of pigs and cause a severe mucohaemorhagic diarrhoea with mucus, fresh blood and/or necrotic material [2, 3].

At the moment, there is no commercial vaccine available for SD. Therefore, the control of this disease relies on the use of a limited number of effective antimicrobials, the most commonly used being the pleuromutilins, tiamulin and valnemulin, macrolides as tylvalosin, and lincosamides as lincomycin [1, 4]. All of these are classified by the European Medicines Agency (EMA) as category C (antibiotics for which alternatives in human medicine generally exist in the European Union (EU) but have to be used with caution in animals). In recent years, the treatment of SD has been hampered by the increase in antimicrobial resistance to the referred antibiotics, as reported in many pig-producing countries [5–7]. Thus, susceptibility testing of clinical isolates recovered from SD outbreaks has become an important need for the swine practitioner.

Among aminoglycosides, gentamicin has been proposed for the treatment and prevention of *Brachyspira* diseases in pigs [2, 8, 9]. Its mechanism of action involves the inhibition of bacterial protein synthesis by binding to the 16S rRNA in the 30S subunit of ribosomes [10]. Nowadays, gentamicin is labelled for the treatment of SD in the United States and several European countries such as Spain and Italy but it has not been widely used for this purpose [2, 9] and there is limited information on *Brachyspira* resistance to this antibiotic. The present study aimed to determine the in vitro susceptibility of *B. hyodysenteriae* field isolates against gentamicin and investigate the molecular mechanisms underlying decreased susceptibility to gentamicin.

## Methods

### Bacterial strains

**Fig. 1:**
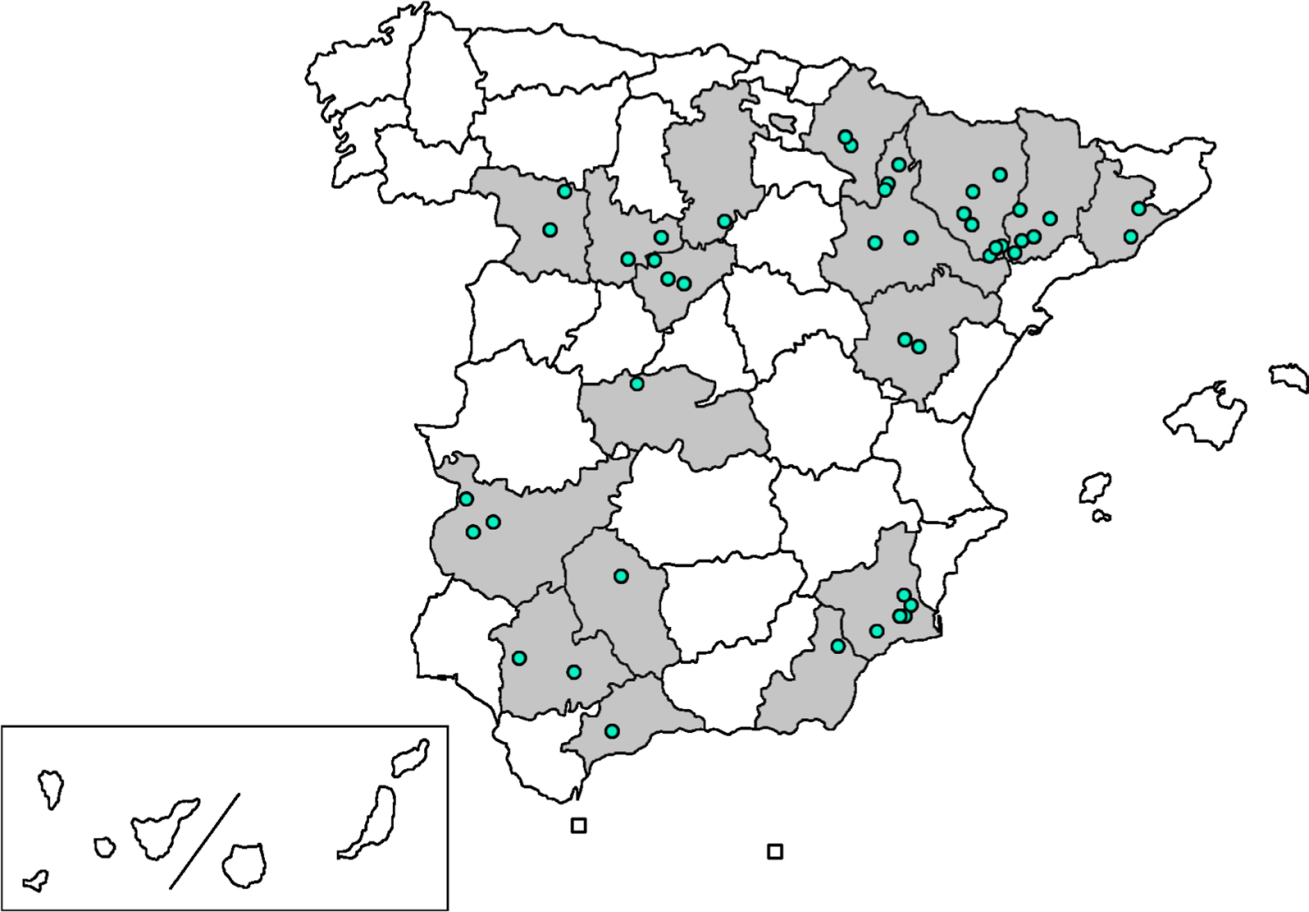
Map showing the distribution of SD outbreaks, between 2011 and 2019, included in this study (shaded areas) and location of sampled farms (blue circles)

A total of 56 *B. hyodysenteriae* field isolates, from the bacterial collection held at the Animal Health Department of the University of León, were used in this study. Isolates were recovered from stool samples of diarrhoea outbreaks that occurred on Spanish pig farms between 2011 and 2019 (one sample per outbreak). Farm locations comprised 17 provinces in the northeast, centre and south of Spain, which represent the most important pig production regions of the country (Fig. 1). *B. hyodysenteriae* reference strain B204 (ATCC 31212) was also included.

### Gentamicin susceptibility testing

Susceptibility testing of each isolate against gentamicin was performed using the broth microdilution method [4, 11] in 48-well tissue culture plates (Iwaki). A stock solution of gentamicin was made by solubilizing gentamicin sulphate salt powder (Sigma-Aldrich) in sterile distilled water to a concentration of 25,600 µg/mL. Plates contained two-fold serial dilutions of gentamicin (128 to 2 µg/mL) made in Brain Heart Infusion Broth (Oxoid) supplemented with 10% of Foetal Bovine Serum (Gibco). For each isolate, a suspension was prepared from a 3-days old culture on tryptic soy agar (TSA) with 5% sheep blood plates (Oxoid) and added to each well reaching a final concentration of approximately 5 × 10^5^ colony forming units (CFU)/mL, based on cell counting with Neubauer chamber. All isolates were tested in triplicate. Positive and negative controls that only contained broth with or without the inoculum, respectively, were included. The plates were incubated in anaerobic boxes with AnaeroGen sachets (Oxoid) for 4 days at 41.5 °C and continuous agitation at 70 rpm. The growth in positive controls was checked for purity by phase-contrast microscopy. Gentamicin activity was determined by MIC, which was defined as the lowest concentration of the antibiotic that inhibited visible growth.

### Monitoring changes in gentamicin susceptibility over time

A survival analysis was conducted to study temporal trends in susceptibility to gentamicin, as previously described [6, 12]. Inhibition of bacterial growth was set as the event and the concentration of antibiotic to the event was analysed instead of time to the event. This approach enables the detection of changes in growth inhibition over the entire range of concentrations. Survival curves were plotted using the nonparametric Kaplan–Meier method. For a clearer graphical representation, twofold serial dilutions of the antimicrobial were log2 transformed.

### Molecular basis of differences in susceptibility to gentamicin

Potential molecular determinants of resistance in the 16S rRNA gene were evaluated by Sanger sequencing. Briefly, DNA from four isolates with no gentamicin resistance (MIC ≤ 2 µg/mL) and five isolates with MIC ≥ 16 µg/mL was extracted by a freeze–thaw cycle and used for the PCR amplification of the complete sequence of the 16S rRNA gene (~ 1500 bp). Each PCR reaction was performed using the DreamTaq DNA polymerase kit (Thermo Scientific) with 20 pmol of primer 27F (5ʹ-AGAGTTTGATCMTGGCTCAG-3ʹ), 20 pmol of primer 1492R (5ʹ-GGTTACCTTGTTACGACTT-3ʹ) and 1 µL of DNA in a final volume of 50 µL. The reaction mixture was subjected to a protocol of amplification in a ProFlex PCR System thermal cycler (Applied Biosystems) that consisted of an initial step of 95 °C for 5 min followed by 35 cycles of 95 °C for 30 s, 57 °C for 30 s and 72 °C for 1 min, with a final extension step of 72 °C for 10 min.

The resulting amplicons were purified with the NucleoSpin Gel and PCR Clean-up kit (Macherey–Nagel) and sequenced in both directions employing the BigDye Terminator v3.1 Cycle Sequencing kit (Applied Biosystems) in an ABI 3500 automated capillary sequencer (Applied Biosystems). Forward and reverse sequences were assembled into contigs and aligned together with the ClustalW tool in MEGA software version 11 [13].

### Association between susceptibility to gentamicin and other antimicrobials

Antimicrobial susceptibility profiles for all *B. hyodysenteriae* isolates were determined prior to this study using commercial plates (VetMIC-Brachy, SVA), which included the six most commonly prescribed antibiotics for the control of SD: tiamulin, valnemulin, tylosin, tylvalosin, doxycycline and lincomycin. Isolates were classified as susceptible or resistant for each antimicrobial using previously proposed clinical breakpoints (Additional files 1 and 2: Tables S1 and S2). MIC_50_ values for gentamicin were estimated among susceptible and resistant isolates to each of the antimicrobials previously tested.

### Isolate typing by multiple-locus variable number tandem-repeat analysis (MLVA)

Four isolates susceptible to gentamicin (2632, 2645, H809 and H862) and five resistant isolates (2611, 6 M, IT-1, IT-18 and H811) were typed by MLVA to study their phylogenetic relatedness. MLVA was performed similarly to previous studies [14]. In brief, the primer pairs used in the individual PCRs were grouped into two sets (set 1 and set 2); labelled fluorescently with 6-carboxyfluorescein (6-FAM), VIC, PET, or NED (Applied Biosystems) at the 5′ end of the forward primers; and pooled as indicated below prior to performing a multiplex PCR using the Qiagen Multiplex PCR kit according to the manufacturer’s recommendations (Qiagen). Primer set 1 was composed of Bhyo_7 (6-FAM), Bhyo_12 (VIC), Bhyo_17 (NED), and Bhyo_22 (PET). Primer set 2 included Bhyo_6 (6-FAM), Bhyo_10 (PET), Bhyo_21 (VIC), and Bhyo_23 (NED). Primer concentrations and PCR conditions were as described elsewhere [14]. GeneScan analysis was performed using an ABI 3730 DNA analyser (Applied Biosystems). The freely available program Peak Scanner Software v1.0 (Applied Biosystems) was used to size the PCR fragments for each locus. The number of repeats was calculated according to the following formula: Number of repeats = [Fragment size (bp) − Flanking regions (bp)]/Repeat size (bp). The results were approximated to the nearest lower integer and sequentially scored (Bhyo_6, Bhyo_7, Bhyo_10, Bhyo_12, Bhyo_17, Bhyo_21, Bhyo_22, and Bhyo_23).

### Statistical analysis

A log-rank test (Mantel-Cox) was performed, at α = 0.05, to compare survival curves from 2011 to 2013 to those from 2014 to 2016 and 2017 to 2019. The Chi-square statistic, at α = 0.05, was used to compare the proportions of gentamicin susceptible and resistant isolates, according to the breakpoints proposed in this work, within isolates classified as susceptible and resistant to each antimicrobial. All analyses were done using the IBM SPSS Statistics software package for Windows, version 25.0 (IBM Corp., Armonk, NY, USA).

## Results

### Gentamicin susceptibility testing

**Fig. 2:**
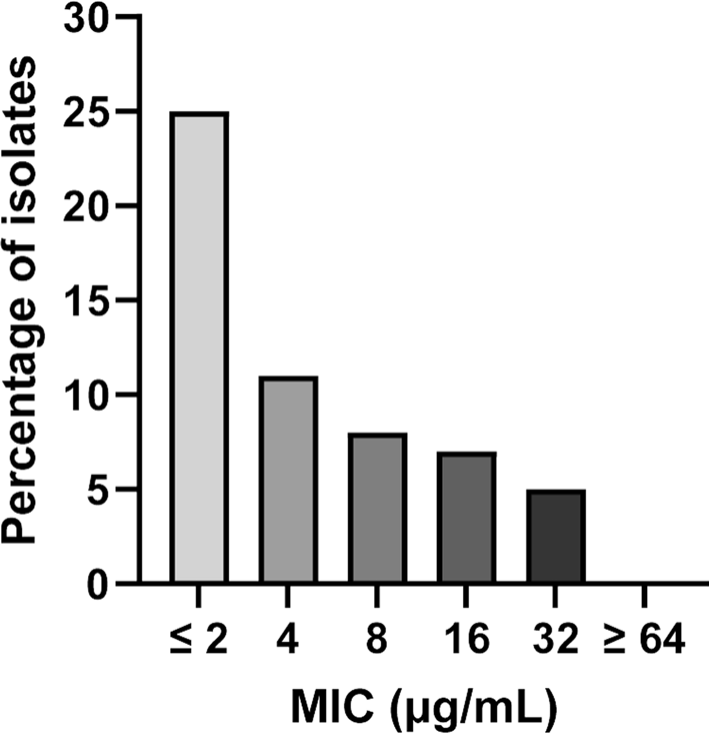
Distribution of gentamicin MICs for 56 Spanish field isolates of *B. hyodysenteriae* recovered between 2011 and 2019

Overviews of the MICs obtained for the 56 *B. hyodysenteriae* isolates tested are shown in Table 1 and Fig. 2. All MIC values for gentamicin ranged between ≤ 2 and 32 µg/mL, with the lowest concentration assessed as the most frequent result and no isolate showing a MIC greater than or equal to 64 µg/mL. Moreover, the values of MIC_50_, the concentration required to inhibit the growth of 50% of the isolates, and MIC_90_, the concentration required to inhibit the growth of 90% of the isolates, were 4 and 16 µg/mL respectively.

**Table 1:** Gentamicin MICs for 56 isolates of *B. hyodysenteriae* recovered between 2011 and 2019 in SD outbreaks on Spanish pig farms

MIC values (µg/mL)	Overall (n = 56)	Time period		
		2011–2013 (n = 19)	2014–2016 (n = 14)	2017–2019 (n = 23)
Minimum MIC	≤ 2	≤ 2	≤ 2	≤ 2
Maximum MIC	32	16	16	32
MIC_50_	4	4	2	4
MIC_90_	16	10.4	8	32
MIC mode	2	2	2	2

### Monitoring changes in gentamicin susceptibility over time

**Fig. 3:**
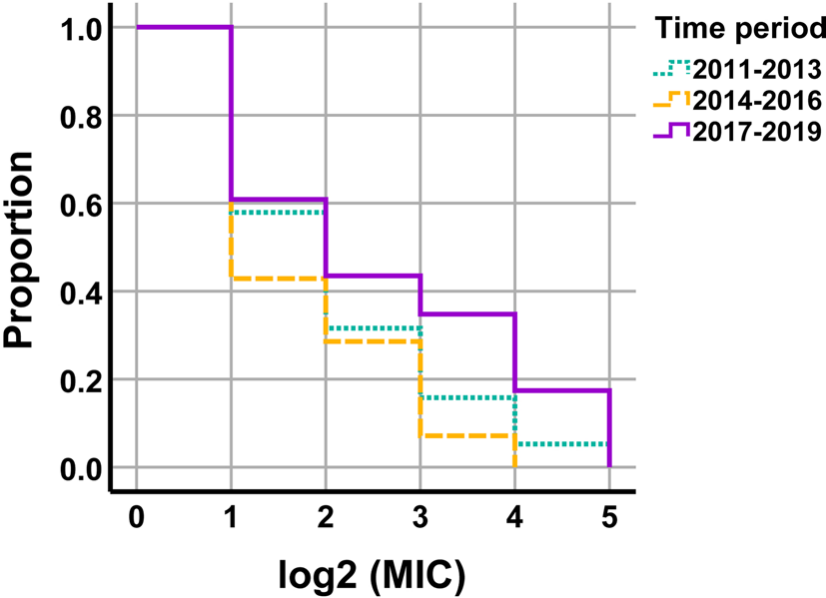
Survival curves of the log_2_ MIC values of gentamicin for 56 Spanish field isolates of *B. hyodysenteriae* recovered between 2011 and 2019

The distribution of MIC values over the period studied (2011–2019) was also analysed. The mode remained constant throughout the study and no progression of MIC_50_ was observed although MIC_90_ increased in the last triennium (Table 1). The survival curves for the three tested periods (2011–2013, 2014–2016 and 2017–2019) are displayed in Fig. 3. The log-rank test demonstrated no statistically significant differences when comparing the survival curve from 2011 to 2013 to those from 2014 to 2016 (*p* = 0.420) and 2017 to 2019 (*p* = 0.226). Differences were close to statistical significance when comparing the curves from the second and the third period (*p* = 0.062).

### Molecular basis of differences in susceptibility to gentamicin

The complete sequence of the 16S rRNA gene was determined in two groups of *B. hyodysenteriae* isolates, which were defined based on their high (n = 5) or low (n = 4) MIC values for gentamicin. Both subsets covered the entire period studied (2011–2019) as well as different geographical origins. The phylogenetic relationship among these isolates was analysed through MLVA typing. The analysis of eight variable number of tandem repeat regions (VNTRs) clustered the nine isolates into four different MLVA types (Table 2).

**Table 2:** MLVA typing results of the isolates selected for gentamicin susceptibility genetic analyses (n = 9) by the analysis of variability in eight VNTR regions

Isolate	VNTR locus								MLVA type
	VNTR-6	VNTR-7	VNTR-10	VNTR-12	VNTR-17	VNTR-21	VNTR-22	VNTR-23	
6 M	1	5	2	3	1	2	3	1	I
IT-1	1	5	2	3	1	2	3	1	I
IT-18	1	5	2	3	1	2	3	1	I
2611	1	4	–	2	4	5	2	1	II
2632	4	1	3	2	2	5	2	1	III
2645	4	1	3	2	2	5	2	1	III
H809	4	1	3	2	2	5	2	1	III
H862	4	1	3	2	2	5	2	1	III
H811	4	4	–	3	2	6	2	1	IV

The observed variations in the nucleotide sequence for each isolate are summarized in Table 3 together with its corresponding gentamicin MIC value and MLVA type. Isolates belonging to subset A exhibited one or two variations in the consensus sequence while, in subset B, each isolate presented at least two and up to seven modified nucleotide positions. None of the recorded variations were common to both subsets. Two substitutions were found in all the isolates belonging to subset B. The first substitution was a C to T transition at the position homologous to 990 in *E. coli* and the second one was an A to G transition at position 1185. Isolates IT-1 and IT-18 additionally shared a C to G transversion at position 1058. All isolates from subset A (sensitive to gentamicin) exhibited the same MLVA type despite differences observed in the gene sequence, while the five isolates from subset B were clustered in three different MLVA groups.

**Table 3:** Gentamicin MICs, MLVA types and nucleotide variations in complete sequences of 16S rRNA gene for susceptible and resistant *B. hyodysenteriae* field isolates

Isolates		MLVA type	MIC (µg/mL)	16S rRNA gene sequence variations^*^										
				C214	C248	C291	C779	T838	C990	C1023	C1058	A1185	C1200	G1206
B204		-	16						T			G	G	
Subset A: susceptible	2632	III	≤ 2			T								A
	2645	III	≤ 2			T								A
	H809	III	≤ 2				T							
	H862	III	≤ 2				T							
Subset B: resistant	2611	II	16						T			G		
	6 M	I	32						T			G		
	IT-1	I	32						T		G	G		
	IT-18	I	32						T		G	G		
	H811	IV	32	T	T			C	T	T		G	A	

^*^ Nucleotide positions according to *Escherichia coli* numbering

### Association between susceptibility to gentamicin and other antimicrobials

The MIC_50_ values for gentamicin were determined in isolates classified as sensitive and resistant to each antibiotic (Table 4). While resistance to lincomycin, tylvalosin or doxycycline was not related with differences in gentamicin MIC values, resistance to pleuromutilins was associated with a lower MIC for gentamicin. Thus, 73% of tiamulin resistant isolates and 20% of tiamulin sensitive isolates were susceptible to gentamicin. Likewise, 83.3% of valnemulin resistant isolates and 33.3% of valnemulin sensitive isolates were classified as susceptible to gentamicin. Based on Chi-square test, the association was close to significance for tiamulin (Chi^2^ = 3.64; *p* = 0.056) and statistically significant for valnemulin (Chi^2^ = 4.25; *p* = 0.039).

**Table 4:** MIC_50_ values of gentamicin in *B. hyodysenteriae* field isolates classified as susceptible or resistant to antimicrobials commonly used for the treatment of SD

Gentamicin MIC_50_ (µg/mL)		
Tiamulin	Susceptible	16
	Resistant	2
Valnemulin	Susceptible	12
	Resistant	2
Lincomycin	Susceptible	2
	Resistant	2
Tylvalosin	Susceptible	4
	Resistant	4
Doxycycline	Susceptible	8
	Resistant	6

## Discussion

Resistance to the main antibiotics registered for the control of SD complicates the management of this disease on swine farms and makes antimicrobial susceptibility testing an essential tool for rational antimicrobial treatment [5]. For this purpose, a commercial plate (VetMIC-Brachy, SVA) which includes six antibiotics, tiamulin, valnemulin, tylosin, tylvalosin, doxycycline and lincomycin, is commonly used for resistance determination in *Brachyspira* species in different countries [5, 15]. Although gentamicin is registered for the treatment of SD in several countries such as Spain (2,000 international units (IU) per kg of body weight during 3 days in drinking water according to the Spanish technical sheet), its use for this purpose is still limited and the information regarding resistance in field isolates is scarce. Considering that the susceptibility to tylosin is almost negligible [4, 5], tylosin could be replaced by gentamicin in these panels, providing more relevant information about antimicrobial sensitivity of *Brachyspira* isolates. Its potential use is subjected to clinical criteria and antimicrobial responsible use guidelines. Aminoglycosides such as gentamicin are used in the treatment of some resistant bacterial infections in humans and are included by the World Health Organization (WHO) in the list of critically important antimicrobials for human medicine, although they are rarely the only treatment option available [16]. This relevance is considered when classified in veterinary medicine, where gentamicin, like the other antibiotics used in the treatment of SD, is classified as category C (use with caution).

In our investigation, a collection of 56 Spanish field isolates of *B. hyodysenteriae* showed low MIC values for gentamicin, with a MIC_50_ of 4 µg/mL, a MIC_90_ of 16 µg/mL and a MIC mode of 2 µg/mL. These values were similar to those reported in 2011 for isolates from North America (MIC_50_ = 4 µg/mL, MIC_90_ = 8 µg/mL, MIC mode = 4 µg/mL) [8] and in 2018 from Taiwan (MIC_50_ = 2 µg/mL, MIC_90_ = 4 µg/mL, MIC mode = 2 µg/mL) [17] using a similar approach. Whilst the activity of gentamicin and other aminoglycosides decreases significantly in the absence of oxygen due to a reduced uptake by bacteria [18, 19], the low MIC values obtained in our study and in previous ones for *B. hyodysenteriae* isolates suggest that even a low uptake could be enough to make it effective against this bacterial species in the strict anaerobic environment of the large intestine. Assessing the evolution of these values over time is essential for the surveillance of resistance development. In this sense, neither the temporal distribution of MICs nor the survival analysis showed any trend towards the development of resistance to gentamicin in field isolates of *B. hyodysenteriae*.

The interpretation of gentamicin MICs for *Brachyspira* species is complicated due to the scarce information on clinical breakpoints*.* These clinical breakpoints must consider the availability of the molecule in its active form in the lumen of the large intestine. There are studies relating the administered dosages in feed or water of some antibiotics used in the treatment of SD with the concentrations reached in the large intestine [20, 21] although there are no similar studies for gentamicin. Nevertheless, it is well known that absorption and degradation of gentamicin is minimal in the small intestine [10, 22] and hence, a high concentration is expected in the lumen of the pig colon. Duhamel et al. proposed the following MIC breakpoints for the evaluation of gentamicin activity in *Brachyspira* spp. by the agar dilution method: susceptible for MIC ≤ 1 µg/mL, intermediate for MIC = 5 µg/mL and resistant for MIC ≥ 10 µg/mL [23]. CLSI guideline for susceptibility testing of infrequently isolated or fastidious bacteria isolated from animals proposed a breakpoint of ≤ 8 µg/mL to classify *B. hyodysenteriae* isolates as susceptible to gentamicin using the agar dilution method [24]. Taking into account that MICs obtained in broth microdilution assays are usually one dilution step lower than the corresponding values from agar dilution [25, 26], we have classified isolates as gentamicin susceptible if MIC ≤ 2 µg/mL, resistant if MIC ≥ 16 µg/mL and intermediate for MIC values of 4 and 8 µg/mL. Therefore, 44.6% (25/56) of the *B. hyodysenteriae* isolates were identified as susceptible to gentamicin, 33.9% (19/56) had reduced susceptibility and 21.4% (12/56) were resistant.

We further explored the genetic determinants of resistance to gentamicin by the study of two extreme populations. Two nucleotide variations were observed in the 16S rRNA gene for all sequenced isolates classified as resistant to gentamicin. These changes were not detected in any of the low MIC isolates. Within the genus *Brachyspira*, point-mutations in genes encoding ribosomal RNA and ribosomal proteins have been associated with an increased MIC for drugs that act by inhibiting protein synthesis such as macrolides, lincosamides [6, 27], pleuromutilins [28–30] and tetracyclines [31, 32]. However, this is the first time to the authors knowledge that point-mutations in the 16S rRNA gene are proposed as a plausible cause of resistance to gentamicin in *Brachyspira* spp. Genetic changes in the 16S rRNA sequence have been widely described to confer resistance to aminoglycosides including gentamicin in other bacterial species like *Escherichia coli* or *Mycobacterium tuberculosis* [33–35]. Typically, these changes affect the aminoacyl-tRNA recognition site (A-site), where aminoglycosides bind in a pocket formed by the A1408 · A1493 base pair and the bulged nucleotide A1492 leading to inhibition of protein synthesis [33, 36]. However, no differences were found at, or nearby, these positions in our *B. hyodysenteriae* isolates. In the present research, mutations were located at positions 990 (C to T) and 1185 (A to G), more than 400 and 200 nucleotides away from the A-site, respectively. The role of these mutations could therefore involve conformational changes that spatially impair the binding of gentamicin, as has also been suggested elsewhere for mutations in distal regions of the 16S rRNA that affect the decoding process in the A-site [37, 38]. Additionally, two of the gentamicin resistant isolates shared a mutation at 1058 (C to G), a position in which nucleotide transversions have been associated with resistance to doxycycline in *B. hyodysenteriae* [28, 31, 39] and *B. intermedia* [32]. It is worth mentioning that resistance to gentamicin can also arise from other mechanisms not analysed in this study, the most widespread being the enzymatic modification and inactivation of the antibiotic, mediated by aminoglycoside acetyltransferases, nucleotidyltransferases or phosphotransferases. The genes encoding these enzymes have been found on plasmids as well as on chromosomes, and are often part of mobile genetic elements. Less relevant mechanisms include increased efflux, decreased cell wall permeability and posttranscriptional modifications (methylation) of specific residues of the 16S rRNA that prevent the binding of the antibiotic [36, 40].

We finally explored the potential phylogenetic relationships among the isolates included in the gentamicin susceptibility genetic analyses. MLVA typing revealed four different clusters. All susceptible isolates were clustered within the same MLVA type, despite exhibiting two different nucleotide variations in the 16S rRNA sequences evaluated and having different origins. It is worth mentioning that the MLVA type of these susceptible isolates is the most frequently observed type identified in clinical outbreaks in Spain that have been investigated by our laboratory (data not shown). In contrast, the subset of gentamicin resistant bacteria clustered in three different MLVA types with different point-mutations, result which demonstrates the lack of association between phylogenetic relatedness and gentamicin resistance. Further studies with larger datasets are required to establish stronger conclusions.

As diseases caused by *Brachyspira* rely heavily on the use of mechanistically analogous antimicrobials, mainly pleuromutilins, macrolides and lincosamides, which act by binding to the 50S ribosomal subunit, there is a high selection pressure for the development of resistance to these drugs [19]. Accordingly, a high proportion of resistance was reported against the main antimicrobials used for the control of SD among the tested *B. hyodysenteriae* isolates, particularly against the pleuromutilin family. These results are in concordance with a number of longitudinal studies from North America, Europe and Japan, reporting the rise of pleuromutilin resistance in this bacterial species as a major threat to the effective control of SD [6, 41–44]. It is worth noting that, in our research, resistance to pleuromutilins was associated with a high susceptibility to gentamicin. Although further studies are required to confirm this relationship, gentamicin could presumably be a suitable alternative for the treatment of SD in outbreaks caused by tiamulin or valnemulin resistant *B. hyodysenteriae* isolates.

## Conclusions

Our results support the use of gentamicin for the treatment of SD caused by *B. hyodysenteriae,* particularly as an alternative in outbreaks associated with pleuromutilin resistant isolates. Accordingly, gentamicin should be included in the routine evaluation of antimicrobial susceptibility of *Brachyspira* isolates and we propose to improve the commercially available plates including this antibiotic, probably replacing tylosin, which is no longer indicated for the treatment of SD due to resistance. In vivo studies to confirm clinical efficacy of gentamicin in SD outbreaks as well as studies to determine the proportion of gentamicin available in its active form in the lumen of the pig colon after its oral administration, allowing for the establishment of clinical breakpoints, are required.

## Supplementary Information

**Additional file 1: Table S1.** Proposed clinical breakpoints for the classification of *B. hyodysenteriae* isolates as susceptible, intermediate or resistant to the antimicrobials commonly used for the treatment of SD

**Additional file 2: Table S2.** Classification of *B. hyodysenteriae* field isolates as susceptible, intermediate or resistant to the antimicrobials commonly used for the treatment of SD

## Data Availability

Data requests should be directed to the corresponding author.

## Abbreviations

CFU: Colony forming units
CLSI: Clinical and Laboratory Standards Institute
EMA: European Medicines Agency
EU: European Union
IU: International units
MIC: Minimum inhibitory concentration
MLVA: Multiple-locus variable number tandem-repeat analysis
SD: Swine dysentery
TSA: Tryptic soy agar
VNTR: Variable number of tandem repeats
WHO: World Health Organization
